# Sulphamoylated 2-Methoxyestradiol Analogues Induce Apoptosis in Adenocarcinoma Cell Lines

**DOI:** 10.1371/journal.pone.0071935

**Published:** 2013-09-05

**Authors:** Michelle Visagie, Anne Theron, Thandi Mqoco, Warren Vieira, Renaud Prudent, Anne Martinez, Laurence Lafanechère, Annie Joubert

**Affiliations:** 1 Department of Physiology, University of Pretoria, Pretoria, South Africa; 2 Institut Albert Bonniot, CRI INSERM/UJF U823, Team 3 Polarity, Development and Cancer, Rond-point de la Chantourne, La Tronche Cedex, France; University of Pecs Medical School, Hungary

## Abstract

2-Methoxyestradiol (2ME2) is a naturally occurring estradiol metabolite which possesses antiproliferative, antiangiogenic and antitumor properties. However, due to its limited biological accessibility, synthetic analogues have been synthesized and tested in attempt to develop drugs with improved oral bioavailability and efficacy. The aim of this study was to evaluate the antiproliferative effects of three novel *in silico*-designed sulphamoylated 2ME2 analogues on the HeLa cervical adenocarcinoma cell line and estrogen receptor-negative breast adenocarcinoma MDA-MB-231 cells. A dose-dependent study (0.1–25 μM) was conducted with an exposure time of 24 hours. Results obtained from crystal violet staining indicated that 0.5 μM of all 3 compounds reduced the number of cells to 50%. Lactate dehydrogenase assay was used to assess cytotoxicity, while the mitotracker mitochondrial assay and caspase-6 and -8 activity assays were used to investigate the possible occurrence of apoptosis. Tubulin polymerization assays were conducted to evaluate the influence of these sulphamoylated 2ME2 analogues on tubulin dynamics. Double immunofluorescence microscopy using labeled antibodies specific to tyrosinate and detyrosinated tubulin was conducted to assess the effect of the 2ME2 analogues on tubulin dynamics. An insignificant increase in the level of lactate dehydrogenase release was observed in the compounds-treated cells. These sulphamoylated compounds caused a reduction in mitochondrial membrane potential, cytochrome *c* release and caspase 3 activation indicating apoptosis induction by means of the intrinsic pathway in HeLa and MDA-MB-231 cells. Microtubule depolymerization was observed after exposure to these three sulphamoylated analogues.

## Introduction

A promising natural metabolite of estradiol, 2-methoxyestradiol (2ME2) has been identified as a possible anticancer agent. 2ME2 exerts *in vitro* (dose- and cell line-dependent) and *in vivo* antiproliferative, antiangiogenic and antitumor activity [1], [2], [3], [4], [5]. Inhibition of proliferation is due to the occurrence of apoptosis, with 2ME2 pursuing actively proliferating cells and quiescent cells are therefore less affected [2]. 2ME2 may be classed as a spindle poison since it disrupts tubulin dynamics by binding to the colchicine site, resulting in either stabilization of the microtubules at low concentration or inhibition of polymerization at higher concentrations [6]. Phase II clinical trials for 2ME2 (Panzem®) are currently being conducted for treatment of multiple myeloma [7], ovarian cancer [8], glioblastoma multiforme [9], breast- and prostate- cancer [10]. However, due to the limited biological accessibility and fast metabolic 2ME2 breakdown, several promising analogues of 2ME2 have been recently developed [11].

2-Methoxyestradiol-bis-sulphamate is a bis-sulphamoylated derivative of 2ME2 which inhibits steroid sulphatase (STS) activity and shows higher antiproliferative activity [12], [13]. Other analogues of 2ME2 showing promising anticancer activities have also been synthesized. These analogues include methylcoumarin-sulphamate (667 Coumate), 2-methoxyestradiol-sulphamate and a second-generation steroid sulphatase inhibitor STX213 which was synthesized by means of adding a *N*-substituted piperidine-2,6-dione ring [14]. Although 667 Coumate has weak aromatase abilities, it is currently undergoing Phase 1 clinical trials for postmenopausal women with advanced or metastatic breast cancer [15]. 2-Methoxyestradiol-sulphamate exerts antiproliferative and antiangiogenic activity and induces a G_2_/M block [16]. STX213 inhibits the proliferation of estrogen dependent positive and estrogen dependent negative breast cancer cells *in vitro*. The second-generation steroid sulphatase inhibitor STX213 has also been shown to cause regression of nitrosomethylurea (NMU)-induced mammary tumors in rodents [15], [16].

Due to the limited metabolic accessibility and rapid degradation of 2ME2 our research team has designed and synthesized novel 2ME2 analogues based on the findings that sulphamate substations increased oestrogenic bioavailability by avoiding hepatic first-pass hepatic metabolism [17], [18]. Not only did the *in silico*-design approach increase carbonic anhydrase IX binding specifically as it is overexpressed in the tumour micro-milieu, but molecules with increased affinity for the colchicine binding site on microtubules could also be selected. This study analyzed *in vitro* effects of these 2ME2 sulphamoylated compounds on a tumorigenic cell lines and investigated their action mechanism.

## Materials and Methods

### Cell lines

Human epithelial cervical cell line (HeLa) was purchased through Sterilab Services (Johannesburg, South Africa) from American Tissue Culture Collection (ATCC) (Maryland, United States of America).

Cells were grown in RPMI (Separations (Randburg, Johannesburg, South Africa), 10% heat-inactivated fetal calf serum100 U/ml penicillin G, 100 µg/ml streptomycin, and 250 µg/l fungizone. Penicillin G, streptomycin, fungizone and trypsin were obtained from Highveld Biological (Pty) Ltd. (Sandringham, South Africa).

MDA-MB-231 is an estrogen receptor-negative breast adenocarcinoma cell line supplied by Microsep (Pty) Ltd, Johannesburg (South Africa). MDA-MB-231 cells were grown in Dulbecco's minimum essential medium eagle (DMEM) and supplemented with 10% heat-inactivated FCS (56°C, 30 min), 100 U/ml penicillin G, 100 µg/ml streptomycin and fungizone (250 µg/l).

### Reagents

All the required reagents of cell culture analytical grade were purchased from Sigma (St. Louis, United States of America) unless otherwise specified. Mitocapture Mitochondrial Apoptosis Detection Kit and the lactate dehydrogenase kit, Caspase 3 colorimetric kit, Caspase 6 colorimetric kit and Fas Associated Death Domain (FADD)-like interleukin-1beta-converting enzyme (FLICE)/Caspase 8 colorimetric kit were purchased from BIOCOM biotech (Pty) Ltd. (Clubview, South Africa). The Flowcellect cytochrome *c* kit was supplied by Millipore Corporation (Billerica, Massachusetts, USA).

Sulphamoylated analogues of 2ME2 were synthesized by Ithemba Pharmaceuticals (Pty) Ltd (Modderfontein, Gauteng, South Africa) since these compounds are not commercially available [17]. Stock solutions of 2-ethyl-3-*O*-sulphamoyl-estra-1,3,5 (10) 16-tetraene (ESE-16), 2-Ethyl-3-*O*-sulphamoyl-estra-1,3,5 (10) 15-tetraene-3-ol-17one (ESE-15-one), and (8R, 13S, 14S, 17S)-2-ethyl-13-methyl-7, 8, 9, 11, 12,13, 14, 15, 16, 17-decahydro-6H-cyclopenta [*a*] phenanthrane-3, 17-diyl bis (sulphamate) (EMBS) were prepared in dimethyl sulfoxide (DMSO) at a concentration of 10mM and were stored at 4°C. Appropriate controls were used in each experiment. One control composed of cells propagated in growth medium alone. Another control was composed of the growth medium with the vehicle, DMSO that never exceeded 0.05%. In addition, cells were exposed to 2-methoxyestradiol-bis-sulphamate (0.55 μM in the growth medium) and actinomycin D (0.1 μg/ml) respectively as positive controls for apoptosis.

### Methods

#### Cell number determination

Crystal violet is a method used to determine the number of cells by staining the deoxyribonucleic acid (DNA). Gillies *et al*. (1986) used crystal violet to quantify cell number in monolayer cultures as a function of the absorbance of the dye taken up by the cells [19]. A dose-dependent study was chosen with a concentration range of 0.1–25 μM since it was previously observed in our laboratory that this concentration series exerts antiproliferative activity in HeLa cells. Exponentially growing HeLa cells were seeded in 96 well tissue culture plates at a cell density of 5,000 cells per well. Cells were incubated at 37°C in a humidified atmosphere containing 5% CO_2_ for 24 h to allow for attachment. To establish the starting number of cells, a baseline determination was conducted before exposure. Subsequently, the medium was discarded and cells were exposed to the sulphamoylated 2ME2 analogues at a concentration series of 0.1–25 μM for the 24 h at 37°C. Vehicle-treated controls were also included. Cells were fixed with 100 µl of 1% gluteraldehyde (incubation for 15 min at room temperature). Gluteraldehyde was discarded and cells were stained using 100 µl 0.1% crystal violet (incubated at room temperature for 30 min). Excedent crystal violet was discarded and the 96 well plate was submersed under running water. Cells were solubilized using 200 µl 0.2% Triton X-100 and incubated at room temperature for 30 min. Solution (100 µl) was transferred to a new microtitre plate. Afterwards, the absorbance was determined at 570 nm using an EL_x_800 Universal Microplate Reader available from Bio-Tek Instruments Inc. (Vermont, United States of America) [20].

### Cytotoxicity: Lactate dehydrogenase assay

Lactate dehydrogenase (LDH) is a soluble cytosolic enzyme that catalyzes the interconversion of lactate and pyruvate. Cells release LDH during injury or cell damage, following the loss of membrane integrity consequential from either apoptosis or necrosis. LDH activity can therefore be used as an indicator of cell membrane integrity and serves as a general mean to assess for cytotoxicity resulting from exposure to chemical compounds. Cells were seeded in 96 well plates at a cell density of 5,000 cells per well (incubated at 37°C at 5% CO_2_). After 24 h, cells were exposed to 0.1–25 μM sulphamoylated 2ME2 analogues including vehicle-treated controls and incubated for 24 h at 37°C. Subsequently, the 200 μl medium was transferred and centrifuged at 5,000 rpm for 10 min. Supernatant (10 μl) was then transferred to an optimally clear 96 well plate. LDH reaction mix (100 μl mixed according to the supplier's manual instructions) was added to the medium. After 90 min incubation at room temperature, the absorbance was read at 460 nm (reference wavelength of 630 nm) with an EL_x_800 Universal Microplate Reader from Bio-Tek Instruments Inc. (Vermont, United States of America).

### Analysis of cell morphology using transmission electron microscopy

The *in vitro* influence of ESE-15-one, EMBS and ESE-16 on cell morphology was determined after exposure for 24 h using transmission electron microscopy (TEM). Cells were fixed in 2.5% glutaraldehyde-formaldehyde mix and then with 0.5% osmium tetroxide. After each fixation step the samples were rinsed 3 times in 0.0075 M sodium phosphate buffer (pH 7.4). Samples were dehydrated using increasing concentrations of ethanol (30%, 50%, 70%, 90%, and 3×100%) and embedded in Quetol resin, sectioned with a microtome and placed on copper discs. Sections were contrasted with 4% aqueous uranyl acetate and Reynolds' lead citrate and viewed with a JOEL JEM 2100F transmission electron microscope (Electron Microscopy Unit, University of Pretoria, South Africa).

### Mitochondrial membrane potential assay

Mitochondrial integrity was investigated by means of a unique cationic dye, 5,5′,6,6′-tetrachloro-1,1′,3,3′- tetraethylbenzimidazolylcarbocyanine iodide. The mitotracker mitochondrial kit provides quantitive apoptosis information. Reduction of the mitochondrial membrane potential is an early feature of apoptosis which is due to the loss of the electrochemical gradient across the mitochondrial membrane. Cells (500,000) were seeded with an overnight attachment policy. After 24 h of exposure to 0.5 μM of the sulphamoylated 2ME2 analogues, cells were detached using trypsin and centrifuged at 13,000×g. Cells (500,000) were resuspended in 1 ml of diluted Mitocapture solution (1 μl mitocapture: 1 ml pre-warmed incubation buffer), incubated under a humidified atmosphere (37°C, 5% CO_2_) for 20 min and subsequently centrifuged at 500×g. Supernatant was discarded and cells were resuspended in 1 ml of prewarmed incubation buffer (37°C). Cells were analyzed immediately following the above-mentioned step using fluorescence activated cell sorting (FACS, FC500 System flow cytometer, Beckman Coulter South Africa (Pty) Ltd). Apoptotic cells were detected in the fluorescein isothiocyanate (FITC) channel (usually FL1) showing diffused green fluorescence. Healthy cells were detectable in the propidium iodide channel (usually FL2) showing red fluorescence. Data from at least 10,000 cells were analyzed by means of Cyflogic version 1.2.1 software (Pertu Therho, Turko, Finland).

#### Cytochrome *c* release

Further involvement of the intrinsic pathway of apoptosis was investigated by demonstrating the effects of the sulphamoylated compounds on cytochrome *c* release utilizing the FlowCellect cytochrome *c* kit (Millipore Corporation, Billerica, Massachusetts, USA.). This kit utilizes a unique set of buffers that selectively permeabilizes the mitochondria without affecting the mitochondrial membrane. Thus viable cells demonstrate higher levels of cytochrome *c* staining, while apoptotic cells presented with reduced staining intensity due to the release of cytochrome *c* from the mitochondria into the cytoplasm. Cells (500,000) were seeded with an overnight attachment policy. After 24 h of exposure to 0.5 μM of the sulphamoylated 2ME2 analogues, cells were detached using trypsin and centrifuged at 13,000×g. Cells (500,000) were resuspended in PBS, centrifuged and resuspended in permeabilization buffer (according to supplier's instructions) for 10 min on ice. Subsequently cells were centrifuged, resuspended in 100 µl fixation buffer (according to supplier's instructions) for 20 min at room temperature, centrifuged again and resuspended in blocking buffer (150 µl). After another centrifugation step, cells were centrifuged and resuspended in blocking buffer (twice). Anti-cytochrome *c*-FITC (10 µl) was added to each sample and samples were incubated for 30 min at room temperature. Cells were centrifuged and blocking buffer (100 µl) was added. After centrifugation, blocking buffer (200 µl) was added to the samples for analysis via flow cytometry (FC500 System flow cytometer, Beckman Coulter South Africa (Pty) Ltd). Cells presenting with cytochrome *c* release were detected in the fluorescein isothiocyanate (FITC) channel (FL1) showing diffused green fluorescence. Data from at least 10 000 cells were analyzed by means of Cyflogic version 1.2.1 software (Pertu Therho, Turko, Finland).

#### Activation of caspases

Possible activation of caspase 3, -6 and -8 was investigated by means of caspase 3, caspase 6 and FLICE/caspase 8 colorimetric kits, respectively. Cells (1,000,000) were seeded with an overnight attachment policy. After 24 h of exposure to 0.5 μM of the sulphamoylated 2ME2 analogues, cells were detached with trypsin and centrifuged at 13,000× g. Cells (500,000) were resuspended in 50 µl of chilled cell lysis buffer and incubated on ice for 10 min. Cells were centrifuged at 10,000×g for a min. Supernatant was transferred to a fresh tube and put on ice. After determination of the protein concentration using the bicinchoninic acid (BCA) protein assay (Thermo Fisher Scientific, Johannesburg, South Africa), 100 µg protein/50 µl cell lysis buffer was mixed with 50 µl 2X reaction buffer (containing 10 mM Dithiothreitol (DTT)). Ac-Asp-Glu-Val-Asp-p-nitroanilide (Ac-DEVD-*p*NA) (5 µl of 4 mM) (caspase-3-specific substrate), or 5 µl 4 mM Ac-Leu-Glu-His-Asp-p-nitroanilide (Ac-VEID-*p*NA) (caspase-6-specific substrate), or 5 µl 4 mM Ac-Ile-Glu-Thr-Asp-p-nitroanilide (Ac-IETD-*p*NA) (caspase-8-specific substrate) was added and the mixture was incubated at 37°C for 120 min (200 µM final concentration). Absorbance was determined at 405 nm on the EL_x_800 Universal Microplate Reader available from Bio-Tek Instruments Inc. (Vermont, United States of America).

#### Tubulin polymerization assay

Microtubule protein (MTP) and pure tubulin were prepared according standard procedures as described by Paturle-Lafanechère *et al*. 1991 [21]. Microtubule polymerization assay was adapted from Bonne *et al*. 1985 [22]. Briefly, microtubule assembly was conducted in a half area 96 well black plate using a microplate reader FLUOstar OPTIMA (BMG Labtechnologies). Wells were charged with either MTP or pure tubulin (final concentration of 25 µM and 30 µM, respectively) in MME (100 mM MES, 1 mM MgCl_2_, 1 mM EGTA, pH 6.75; for MTP) or PME (100 mM PIPES, 1 mM MgCl_2_, 1 mM EGTA, pH 6.65; for pure tubulin) buffer with 10 µM DAPI and variable concentrations of compounds to be assayed. Following 10 min incubation, assembly was initiated by injection of GTP and MgCl_2_ to a final concentration of 1 mM and 5 mM respectively, yielding a reaction volume of 100 µl. The excitation and emission wavelengths were set at 360 and 450 nm, respectively, and the fluorescence of microtubule-bound DAPI was monitored as a function of time at 37°C. Fluorescence signal at time 0 for each well was subtracted from each of the subsequent fluorescence readings [21], [22].

#### Double immunofluorescence for microscopic analysis of intracellular microtubules

In order to visualize the effect of the 2ME2 analogues on microtubule dynamics and integrity, a double immunofluorescence technique using antibodies against tyrosinated and detyrsosinated tubulin was conducted as described by Paturle-Lafanechère *et al.* (2004) [23]. HeLa and MDA-MB-231 cells were seeded onto sterilized coverslips and allowed to grow to 80% confluency for 48 h. Cells were exposed to 0.189 μM ESE-15-one, 0.5 μM EMBS and ESE-16 along with DMSO (negative control) and 1μM colchicine as a positive control for 24 h. Cells were permeablised permeabilized with warmed OPT buffer (80 mmol/L Pipes, 1 mol/L EGTA, 1 mol/l MgCl_2_, 0.5% Triton X-100 and glycerol 10%, pH 6.8), followed by fixation in ice-cold methanol at −20°C and were subsequently incubated with the primary antibody cocktail (PBS containing anti-detyrosinated tubulin (L4), anti-detyrosinated antibody YL1/2, BSA 0.3%, tween 0.1%) [24]. After incubation, cells were incubated with the secondary antibodies, namely Alexa 488 (Invitrogen) and Cy3 (Jackson ImmunoResearch laboratories). Samples were visualized using a Zeiss AxioImager Z1 microscope controlled by Axiovision softwared (Carl Zeiss) (X63 oil objective). Images were captured using an Ocra R2 N/B camera (Hamamatsu) and assessed for microtubule integrity and ratio of tyrosinated to detyrosinated tubulin.

#### Statistical analysis of data

The ANOVA students'*t*-test was used to determine the analytical variation in experimental procedures and biological variations within each experiment. Cell growth studies were repeated three times with a sample size of 6 in each experiment. Means are presented in bar charts, with T-bars referring to standard deviation. A *P*-values of <0.05 was regarded as statistically significant (indicated by an asterisk; *). Flow cytometry results were analyzed from 10 000 events.

## Results

### Cell number determination

**Figure 1:**
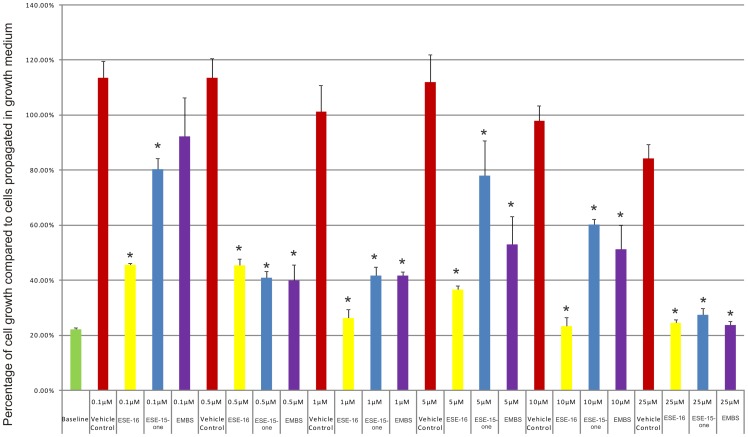
Cell number determination. HeLa cell numbers expressed as a % of cells relative to the control (cells propagated in growth medium) after exposure to ESE-15-one, EMBS and ESE-16 for 24 h. An asterisk (*) indicates a statistically significant *P*-value of <0.05 when compared to cells propagated in growth medium.

Dose-dependent studies were conducted with the purpose of evaluating the antiproliferative effects of ESE-15-one, EMBS and ESE-16 in HeLa cells after 24 h of exposure. As previously mentioned, a dosage range of 0.1–25 µM was chosen since it was previously found that the compounds caused a decrease in cell growth in this concentration range. ESE-15-one (0.1 µM) statistically significantly reduced cell growth to 80.31% and 0.1 µM ESE-16 statistically significantly reduced cell growth to 45.59%. Growth (92.32%) of EMBS-treated cells was not statistically significantly inhibited (Fig. 1). A statistically significant decrease in cell growth was observed at a concentration of 0.5 µM with ESE-16 (45.30% growth), ESE-15-one (40.87% growth) and EMBS (40.01% growth) when compared to vehicle-treated cells. Cells exposed to 5 µM and 10 µM of ESE-15-one and EMBS revealed a slight decrease in cell growth (Fig. 1). This observed biphasic effect is also a characteristic of 2ME2. Cells treated with 25 µM of all compounds respectively displayed a pronounced reduction in cell growth. Cells exposed to ESE-16 demonstrated the most prominent decrease in cell growth when compared to cells exposed to ESE-15-one and EMBS. The concentration of ESE-15-one, EMBS and ESE-16 that significantly inhibits HeLa proliferation after 24 h of exposure was found to be 0.5 µM. This concentration was thus chosen as the dose of exposure for subsequent experiments.

### Cytotoxicity: Lactate dehydrogenase assay

**Figure 2:**
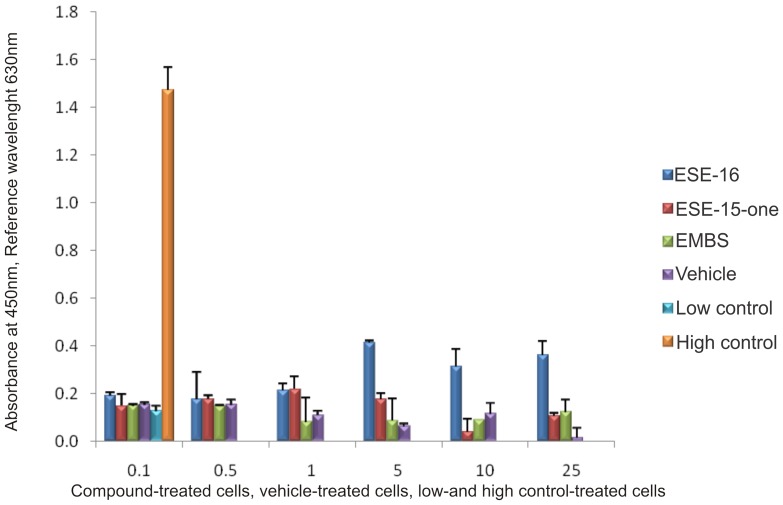
Cytotoxicity by means of lactate dehydrogenase assay. LDH levels of ESE-15-one, EMBS and ESE-16 -treated HeLa cells and vehicle-treated control cells after 24 h exposure. The background control consists of growth medium only. The low control refers to cells resuspended in growth medium and the high control to cells resuspended in growth medium with cell lysis solution added to the cells shortly before the experiment was terminated (according to the manufacturer's instructions). No statistically significant increase in LDH levels was observed in treated cells. These results showed that the sulphamoylated compounds were not toxic to the cells.

The LDH assay was conducted to measure cytotoxicity of ESE-15-one, EMBS and ESE-16. A statistically insignificant increase in LDH levels was observed in the compounds-treated cells when compared to the vehicle-treated cells. ESE-16-treated cells revealed the largest increase in LDH release when compared to vehicle-treated cells and cells treated with ESE-15-one and EMBS (Fig. 2).

### Analysis of cell morphology using transmission electron microscopy

**Figure 3:**
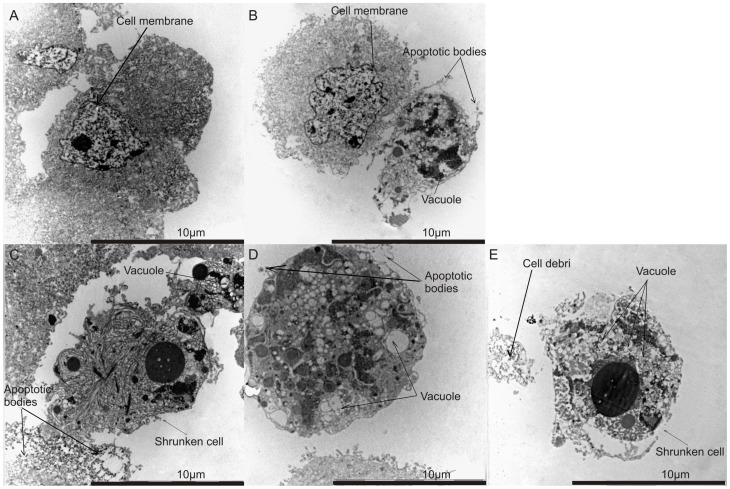
Morphology investigation using transmission electron microscopy. TEM revealed vehicle-treated cells displaying no signs of distress (A). Cells treated with 2-methoxyestradiol-bis-sulphamate (B), ESE-16 (C) and ESE-15-one (D) demonstrated the occurrence of apoptotic bodies and vacuole formation. Cells treated with EMBS (E) displayed an increase in the number of vacuoles.

Transmission electron microscopy revealed vacuoles in cells treated with ESE-15-one, EMBS, ESE-16 and 2-methoxyestradiol-bis-sulphamate. The latter was used as a positive control for apoptosis and autophagy (Fig. 3). Increased occurrence of vacuoles is indicative of cell death through autophagy. Apoptotic bodies were observed in ESE-15-one-treated cells, ESE-16-treated and cells treated with 2-methoxyestradiol-bis-sulphamate. These apoptotic bodies were not detected in vehicle-treated cells. Cell debris was observed in EMBS-treated cells. In addition, cells appeared shrunken in ESE-16-treated cells, ESE-15-one-treated cells and EMBS-treated cells when compared to vehicle-treated cells. These morphological changes observed in treated cells are hallmarks of apoptosis.

### Mitochondrial membrane potential assay

**Figure 4:**
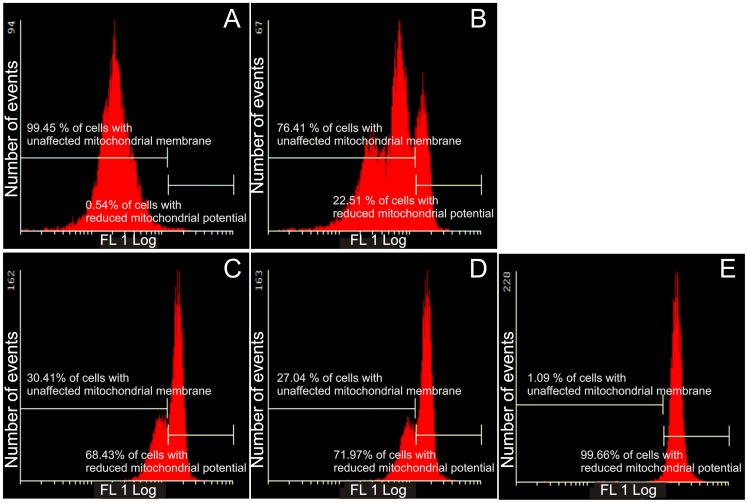
Mitochondrial membrane potential assay of HeLa cells. Mitotracker-stained vehicle-treated control cells (A), 2-methoxyestradiol-bis-sulphamate-treated cells (B), ESE-16-treated cells (C), ESE-15-one-treated cells (D) and EMBS-treated cells (E) after 24 h exposure. An increase in the number of cells with reduced mitochondrial potential treated with 2ME2 analogues compared to the vehicle-treated control cells was observed.

**Figure 5:**
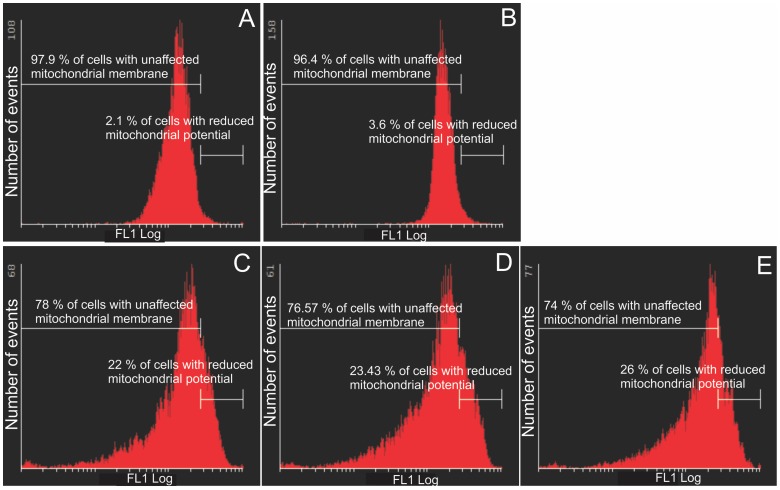
Mitochondrial membrane potential assay of MDA-MB-231 cells. Mitocapture-stained vehicle-treated control cells (A), 2-methoxyestradiol-bis-sulphamate-treated cells (B), ESE-16-treated cells (C), ESE-15-one-treated cells (D) and EMBS-treated cells (E) after 24 h exposure. An increase in the number of cells presenting with compromised mitochondrial potential was demonstrated in all three compound-treated cells when compared to the vehicle-treated control cells.

One of the earliest intracellular events that occur following the induction of apoptosis is the reduction of the mitochondrial transmembrane potential. The Mitocapture assay was used to investigate possible induction of apoptosis by 2ME2 analogues (Fig. 4 and Fig. 5). The exposure of HeLa cells to 2ME2 analogues resulted in a statistically significant increase in the number of cells with a reduced mitochondrial membrane potential. ESE-16-treated cells (68.43%) exhibited reduced mitochondrial potential and 72% ESE-15-one-treated cells presented with reduced mitochondrial potential. Cells exposed to EMBS were the most prominently affected demonstrating 98.66% of cells having reduced mitochondrial membrane potential. Exposure of MDA-MB-231 cells to sulphamoylated compounds resulted in a significantly increased number of cells presenting a reduced mitochondrial membrane potential (22–26%), although the effect was less prominent than for HeLa cells.

### Cytochrome *c* release

**Figure 6:**
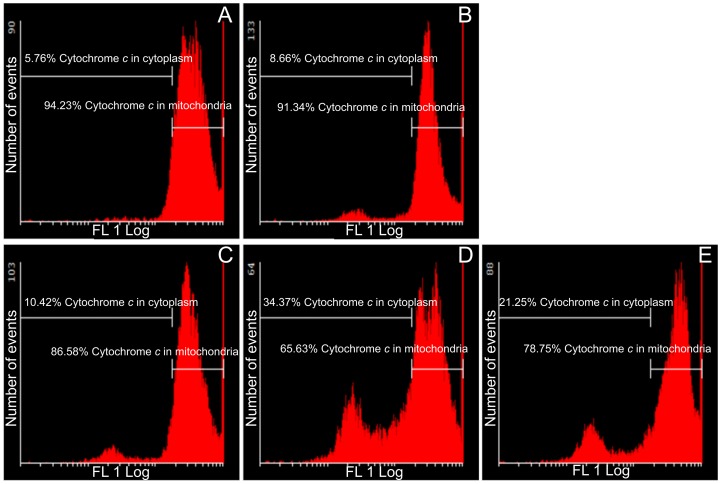
Assay demonstrating cytochrome *c* release in HeLa cells. Flow cytometry was used to show the effects of these compounds on cytochrome *c* release from the mitochondria into the cytoplasm in HeLa cells. Vehicle-treated control cells (A), 2-methoxyestradiol-bis-sulphamate-treated cells (B), ESE-16-treated cells (C), ESE-15-one-treated cells (D) and EMBS-treated cells (E) following 24 h of exposure. Cytochrome *c* release was evident in all three compound-treated cells when compared to the vehicle-treated cells.

**Figure 7:**
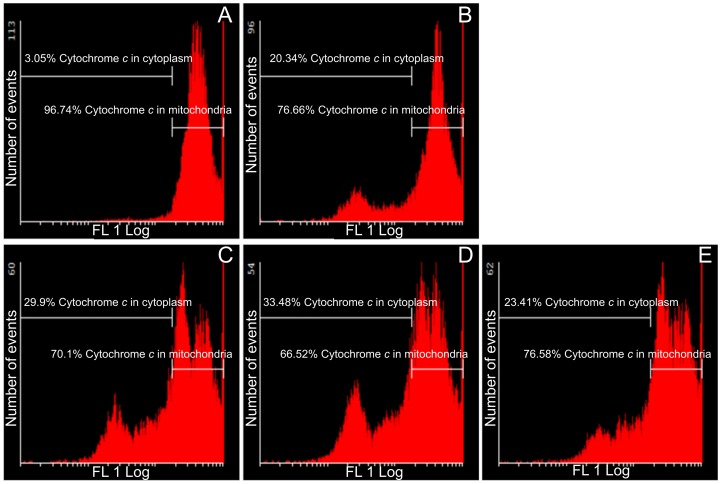
Assay demonstrating cytochrome *c* release in MDA-MB-231 cells. Flow cytometry was conducted to present the effects of these sulphamoylated compounds on cytochrome *c* release from the mitochondria into the cytoplasm. Vehicle-treated control cells (A), 2-methoxyestradiol-bis-sulphamate-treated cells (B), ESE-16-treated cells (C), ESE-15-one-treated cells (D) and EMBS-treated cells (E) following 24 h of exposure. All three compound-treated cells demonstrated increased levels of cytochrome *c* release when compared to the vehicle-treated cells.

Further involvement of the intrinsic apoptotic pathway was investigated by means of demonstrating the effects of these sulphamoylated compounds on cytochrome *c* release (Fig. 6 and Fig. 7). Flow cytometry data indicated that all sulphamoylated compounds have a statistically significant effect on both cell lines with regard to cytochrome *c* release from the mitochondria into the cytosol. ESE-15-one-treated HeLa (35% of cells presenting with cytochrome *c*) and MDA-MB-231 cells (34% of cells presenting with cytochrome *c*) were prominently affected. ESE-16-treated cells presented with cytochrome *c* release from mitochondria into the cytosol in MDA-MB-231 cells (30%) and HeLa cells (10%). EMBS-treated HeLa and MDA-MB-231 cells presented with 21–23% cytochrome *c* release from mitochondria into the cytosol.

### Possible activation of caspases

**Figure 8:**
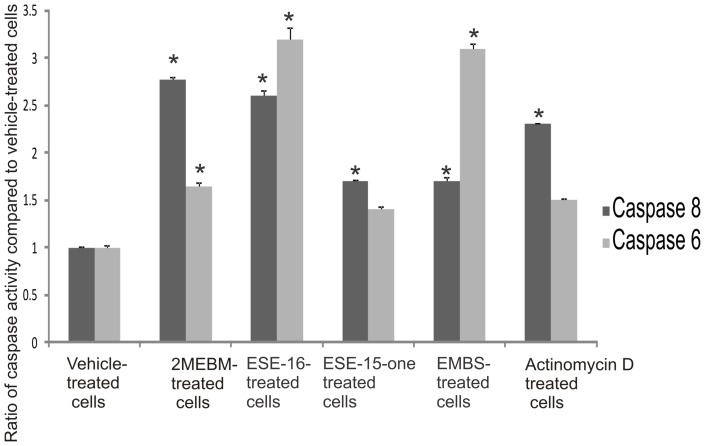
Determination of caspase activation. Caspase 8 and caspase 6 activity ratios of compound- and actinomycin D-treated cells compared to vehicle-treated cells. Caspase 6 and caspase 8 activities in all compound-treated cells increased when compared to vehicle-treated cells. EMBS-treated cells demonstrated the most prominent increase in caspase 8 activity and ESE-16-treated cells the most prominent increase in caspase 6 activity when compared to vehicle-treated cells. 2-Methoxyestradiol-bis-sulphamate-treated cells are illustrated in the figure as 2MEBM due to limited space. An asterisk (*) indicates a statistically significant *P*-value of <0.05 when compared to vehicle-treated cells.

**Figure 9:**
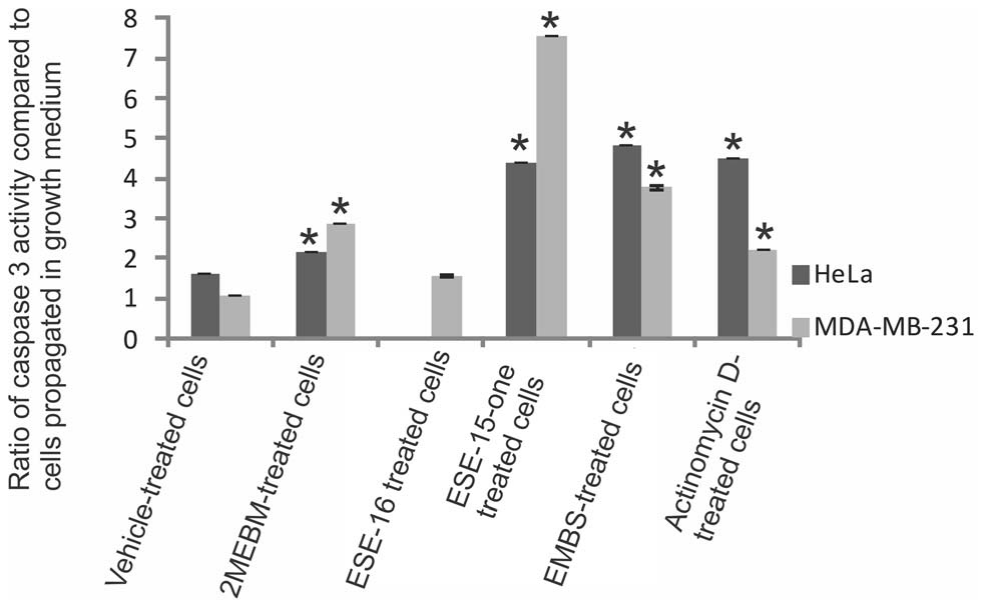
Caspase 3 activity in HeLa and MDA-MB-231 cells. Spectrophotometry results of caspase 3 activity indicated that after exposure to these sulphamoylated compounds caspase 3 activity increased significantly. An asterisk (*) indicates a statistically significant *P*-value <0.05 when compared to cells propagated in growth medium.

Investigation of possible induction of caspase activity was conducted by means of a caspase-6 and -8 colorimetric assays. Data indicated that there was an increased caspase-8 activity in ESE-15-one, EMBS and ESE-16-treated cells when compared to vehicle-treated cells (Fig. 8). ESE-15-one and ESE-16-treated cells displayed 1.7× fold increase. Cells treated with EMBS demonstrated the most prevalent increase (2.6-fold) when compared to the other compound-treated cells. EMBS-treated cells revealed increased caspase-8 activity comparable to that of cells treated with 2-methoxyestradiol-bis-sulphamate and actinomycin D. Caspase-6 colorimetric studies demonstrated an increased caspase-6 activity in compound-treated cells when compared to vehicle-treated cells (Fig. 8). Caspase-6 activity of cells treated with ESE-16 increased to 3.2 and EMBS to 3.1. Caspase 3 activation by sulphamoylated compounds in the cervical tumorigenic and estrogen receptor-negative cell lines was demonstrated using a colorimetric assay (Fig. 9). Caspase 3 activity in ESE-15-one- and EMBS-treated samples more than doubled when compared to vehicle-treated cells. In another study conducted in our laboratory caspase 3 activity was increased (8-fold) in HeLa cells after exposure to ESE-16 (data not shown). Results obtained from the MDA-MB-231 cell line indicate that all three compounds increase caspase 3 activity with ESE-15-one-treated cells being most affected.

### Tubulin polymerization assay

**Figure 10:**
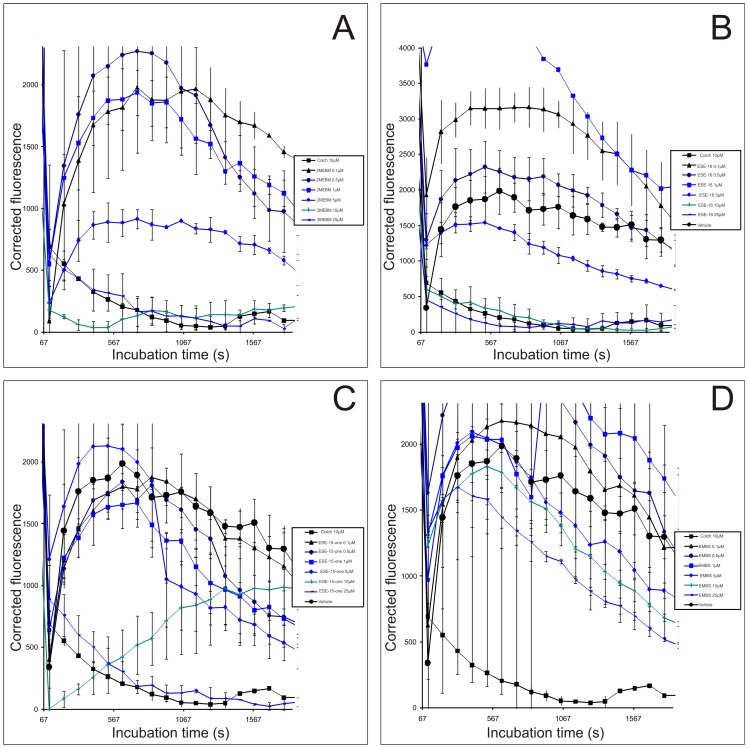
Tubulin polymerization assay. Tubulin was incubated with the compounds ((A), ESE-16 (B), ESE-15-one (C) and EMBS (D)), as described in the methods section. This assay showed that ESE-16 had a pronounced effect comparable with that of colchicine.

The antiproliferative effect of 2ME2 has been shown to stem from its ability to inhibit tubulin assembly by interacting at the colchicine site of tubulin. In order to determine if the synthesized compounds may be active on cell proliferation through the same mechanism of action, the compounds' *in vitro* effects on tubulin polymerization were analyzed (Fig. 10). *In vitro*, the three compounds showed a depolymerizing activity. ESE-16 was shown to exert a more pronounced effect on tubulin polymerization when compared to the other analogues and to colchicine-treated cells.

### Double immunofluorescence microscopy for microtubule dynamics

**Figure 11:**
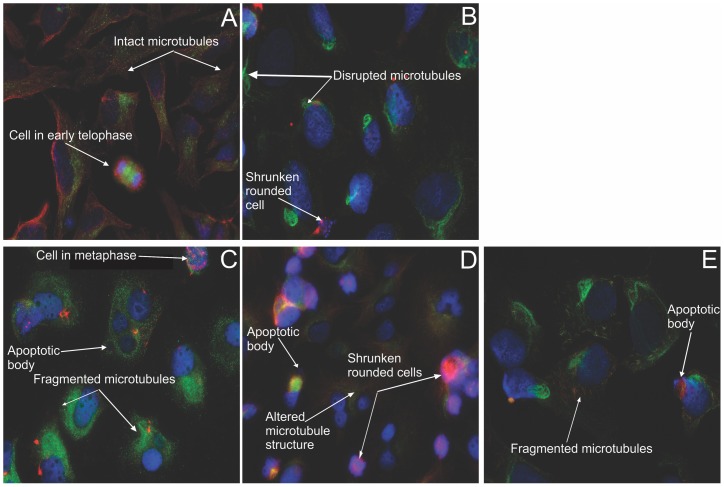
Immunofluorescent determination of microtubule dynamics in HeLa cells. Double immunoflourecence was conducted to determine the compounds' effect after 24 h on microtubule dynamics within HeLa cells. Tyrosinated (dynamic) microtubules are visualized as red, whereas the detyrosinated (stable or stabilized) microtubules are stained in green. The vehicle-treated cells (A) demonstrated an intact dynamic microtubule structure. Both the colchicine positive control (B), as well as the ESE-16-treated cells (C) showed complete microtubule depolymerisation with few detyrosinated microtubule fragments remaining. ESE-15-one-treated cells (D) demonstrated altered microtubule morphology. EMBS-exposed cells (E) revealed fragmented microtubules. All treated cell samples demonstrated a decrease in cell density and rounded cells (X63 oil objective).

**Figure 12:**
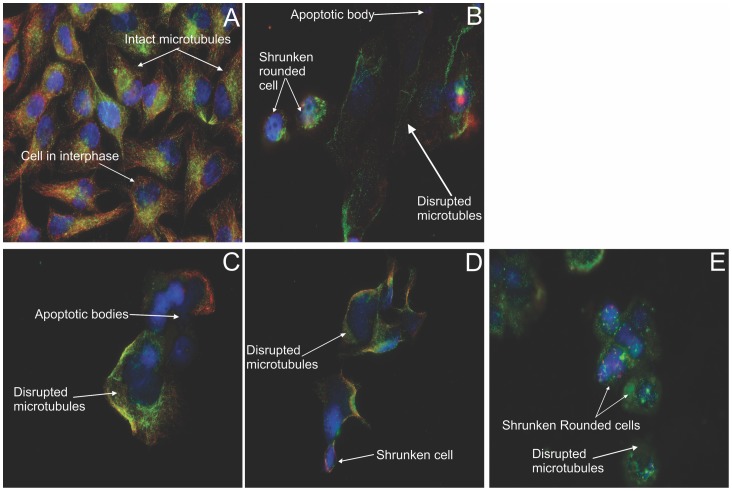
Immunofluorescent determination of microtubule dynamics in MDA-MB-231 cells. Double immunoflourecence was done to determine the compounds' effect after 24 h on microtubule dynamics within MDA-MB-231 cells. Tyrosinated (dynamic) microtubules are visualized as red, whereas the detyrosinated microtubules are stained green. The vehicle-treated cells (A) demonstrated an intact dynamic microtubule structure. The colchicine-exposed positive control cells (B) showed complete microtubule depolymerisation with few detyrosinated microtubule fragments remaining. ESE-16 (C), ESE-15-one (D) and EMBS-treated cells (E) demonstrated detyrosinated microtubule fragments. All treated cell samples demonstrated a decrease in cell density with rounded, shrunken cells. Apoptotic bodies are evident in the ESE-16 treated cells (X63 oil objective).

By employing a method based on the substrate properties of the tubulin enzymes involved in the tubulin tyrosination cycle, dynamic microtubules composed of tyrosinated tubulin, as well as non-dynamic stabilized microtubules (mostly composed of detyrosinated tubulin) can be distinguished via double immunofluorescence [23], [25]. Nuclear counterstaining was done with DAPI. A 24 h exposure of both HeLa (Fig. 11) and MDA (Fig. 12) cells to the compounds was conducted. Vehicle-treated cells possess intact tyrosinated (red) microtubules (Fig. 11A and 12 A). Disruption of microtubule structures was demonstrated in all the other figures. The positive colchicine control (Fig. 11B), displayed enlarged rounded cells, with only fragments of detyrosinated microtubules remaining after 24 h. ESE-15-one-treated cells (Fig. 11D), EMBS-treated cells (Fig. 11E) and ESE-16-treated cells (Fig. 11C) showed microtubule abrogation, with a predominance of residual detyrosinated microtubules, rounded cells and a decreased cell density. Figure 12 represents MDA-MB-231 cells exposed to the analogues, with the same consequence as in the HeLa cells. Theses micrographs suggest that the 2ME2 analogues work in a similar way to colchicine in disrupting tubulin dynamics resulting in a predominantly depolymerized microtubule network and a few residual detyrosinated microtubule fragments, which consequently do not allow the cell to pass the cell cycle checkpoints resulting in apoptotic cell death.

## Discussion

This study focused on investigating the *in vitro* effects of three novel sulphamoylated 2ME2 analogues on tubulin assembly, microtubule dynamics, cell growth, cell toxicity, caspase activity and cell death induction in a cervical tumorigenic cell line. In addition, the involvement of the intrinsic pathway in HeLa and MDA-MB-231 cells induced by these sulphamoylated compounds was demonstrated by investigating their effects on mitochondrial membrane potential, cytochrome *c* release and caspase 3 activation. Crystal violet staining showed that ESE-15-one, EMBS and ESE-16 inhibited cell growth significantly where EMBS demonstrated the most potent effect. Research conducted in our laboratory indicated that other analogues of 2ME2 had an inhibitory effect on cell growth in this range (0.2 μM–1 μM) in several cell lines including the MCF-7 cell line and esophageal carcinoma SNO cells [17], [26], [27], [28], [29]. Furthermore, the second-generation steroid sulphatase inhibitor, STX213, inhibits the proliferation of estrogen dependent positive and estrogen dependent negative breast cancer cells *in vitro* [15], [16]. Antiproliferative activity induced by 2-methoxyestradiol-bis-sulphamate was detected in the estrogen receptor positive human breast adenocarcinoma MCF-7 cell line (0.1–1 μM), prostate cancer cell line (LNACaP), tumorigenic estrogen receptor negative breast adenocarcinoma cell line (MDA-MB-231), esophageal carcinoma SNO cells, HeLa and the CAL51 human breast carcinoma cell line [26], [27], [28], [29], [30], [31], [32], [33]. *In vivo* antiproliferative activity was discovered in xenografts derivative of estrogen receptor positive human breast adenocarcinoma wild type cell line (MCF-7_WT_), mitoxantrone resistant breast adenocarcinoma cell line (MCF-7 MR), drug resistant human adenocarcinoma cell line (MCF-7 _DOX_40), prostate cancer cell line (LNACaP), MDA-MB-435 and prostate hormone independent PC-3 xenograft model [31], [34], [35], [36], [37], [38], [39].

Pertaining to 2ME2 it was found that the sulphamoylated analogues targeted microtubules dynamics, both *in vitro* and in cells. The *in vitro* tubulin polymerization assays indicated that sulphamoylated analogues have increased or similar potencies when compared to 2-methoxyestradiol-bis-sulphamate (EC_50_ = 11 µM). Ranking of compounds according to tubulin polymerization inhibition potency is as follow: ESE-16 (EC_50_ = 5 µM) > EMBS (EC_50_ = 14 µM) >2-methoxyestradiol-bis-sulphamate > EMBS. This indicates that chemical modifications performed to restrict drug metabolism do not impair target binding.

Chemical agents which disrupt microtubule dynamics have been a cornerstone in cancer therapy for many years. Since microtubules play an essential role in cell division and the cell cycle, dynamics blockage halts the cell cycle at the metaphase/anaphase transition due to failure to pass the spindle assembly checkpoint [40], [41]. This consequently results in programmed cell death. Double immunofluorescence microscopy using antibodies directed toward stabilized detyrosinated microtubules and dynamic tyrosinated microtubules allowed a visual analysis of the influence of 2ME2 analogues on microtubule dynamics and integrity in HeLa and MDA-MB-231 cells. The analogue-treated cells revealed a pronounced effect correlating with the *in vitro* purified tubulin dimerization results. A shift toward residual stabilized microtubule fragments, together with abrogation of the microtubule structure on exposure to all the three analogues, with subsequent apoptosis induction in a similar fashion to the colchicine control was observed.

Although studies by Xia *et al*. (2007) and Kachadourian *et al*. (2001) showed that the exposure of MDS-RAEB MUTZ-1 cells (a cell line derived from the bone marrow of an individual with myelodysplastic syndrome) and human leukemia HL-60 cells to 2ME2 resulted in an increase in LDH production [42], [43], it was found that the *in vitro* cytotoxic effect of the sulphamoylated 2ME2 analogues does not reflect possible damage to cell membrane integrity. These investigations did not result in a statistically significant increase in the production of LDH in HeLa cells. This was consistent with previous data from our laboratory, which demonstrated that the exposure of a non-tumorigenic breast cancer cell line (MCF-12A) and esophageal cancer cell line (SNO) to 2-methoxyestradiol-bis-sulphamate resulted in a statistical insignificant release of LDH [27], [28]. This indicated that sulphamoylated 2ME2 analogues do not induce an important severing of HeLa cell membrane.

Exposure of HeLa and MDA-MB-231 cells to the sulphamoylated 2ME2 analogues resulted in a statistically significant increase in the number of cells with reduced mitochondrial membrane potential, cells presenting cytochrome *c* release and caspase 3 activation, indicating apoptosis induction by means of the intrinsic pathway. Foster *et al*. (2008) demonstrated that 500 nM of 2-methoxyestradiol-bis-sulphamate depolarized the inner mitochondrial membrane potential in human breast adenocarcinoma estrogen negative cell line (MDA-MB-231) and human umbilical vein endothelial cells (HUVECs) after 72 hours of exposure [13]. Morphological studies (TEM) demonstrated that the sulphamoylated 2ME2 analogues induced autophagy and apoptosis in HeLa cells. Such an observation was also made in 2-methoxyestradiol-bis-sulphamate exposed MCF-7 [28]. Thus, regarding the effects on mitochondria and the observed morphological changes, sulphamoylated 2ME2 analogues behave in a similar manner to 2ME2.

2-Methoxyestradiol-bis-sulphamate induced apoptosis in MCF-7, MDA-MB-231, prostate cancer cells (PC-3), human umbilical vein endothelial cells (HUVEC), and human breast adenocarcinoma CAL51 cells [44], [45], [46]. Caspases 6 and 8 activity assays were thus conducted to determine which pathway of apoptosis is activated by 2ME2 analogues. Results showed an increase in both caspase 8 and 6 activities in compound-treated cells. The increase in the number of cells accompanied with reduced mitochondrial membrane potential and increased caspase 8 activity suggests that these 2ME2 analogues induces apoptosis by means of both the intrinsic and extrinsic pathways, respectively.

In conclusion, this work has produced evidence that these novel sulphamoylated 2ME2 analogues, ESE-15-one, EMBS and ESE-16 exert antiproliferative effects *in vitro* and induce apoptosis in a human cervical adenocarcinoma cell line with insignificant influences on LDH release. Morphological studies suggested induction of both apoptosis and autophagy as types of cell death, most likely induced by a spindle abrogative effect. Occurrence of apoptosis was confirmed by revealing caspase-6 and 8 activation. Compromised mitochondrial membrane potential, cells presenting cytochrome *c* release and caspase 3 activation after exposure to these three sulphamoylated compounds in the cervical and metastatic cell lines indicate the involvement of the mitochondria and thus the intrinsic pathway resulting in apoptotic cell death. This study resulted in an improved understanding of the cellular effects of the novel sulphamoylated 2ME2 analogues. Future studies are warranted to unravel molecular cross-talk mechanisms between the induced autophagic and apoptotic cell death mechanisms, as well as investigating their *in vivo* efficacy in cancer treatment.
